# In Vitro Efficiency of Antimicrobial Peptides against Staphylococcal Pathogens Associated with Canine Pyoderma

**DOI:** 10.3390/ani10030470

**Published:** 2020-03-11

**Authors:** Małgorzata Jarosiewicz, Katarzyna Garbacz, Damian Neubauer, Wojciech Kamysz

**Affiliations:** 1 Department of Oral Microbiology, Faculty of Medicine, Medical University of Gdansk, Gdansk, 25 Dębowa Str., 80-204 Gdansk, Poland; malgorzata.jarosiewicz@gumed.edu.pl; 2 Department of Inorganic Chemistry, Faculty of Pharmacy, Medical University of Gdansk, Gdansk, 107 Gen. J. Hallera Str., 80-416 Gdansk, Poland; damian.neubauer@gumed.edu.pl (D.N.); wojciech.kamysz@gumed.edu.pl (W.K.)

**Keywords:** antimicrobial peptides, canine pyoderma, *Staphylococcus pseudintermedius*

## Abstract

**Simple Summary:**

Coagulase-positive staphylococci (CoPS) are predominant pathogens in canine pyoderma, especially *S. pseudintermedius* and *S. aureus*. The antimicrobial resistance of CoPS has a key role in the management of canine skin infections. The vast majority of those diseases have a chronic character with a tendency to recur, which is reflected by recurrent systemic antibiotic therapy, associated with an alarming increase in the proportion of antibiotic-resistant staphylococci. Antimicrobial peptides (AMPs) seem to be a promising alternative to conventional antibiotics. The aim of this in vitro study was to evaluate the antimicrobial activity of selected AMPs against pathogenic staphylococcal strains, including multidrug- and methicillin-resistant strains isolated from canine pyoderma cases. The tested AMPs were shown to be equally efficient antimicrobial agents against resistant- and susceptible pathogenic staphylococcal strains associated with canine pyoderma. AMPs were more efficient against *S. pseudintermedius* than against *S. aureus* strains. Our findings seem to be particularly interesting from a clinical perspective, as a starting point from which to perform in vivo experiments to estimate the usefulness of these peptides as topical drug molecules for the treatment of canine pyoderma.

**Abstract:**

The emergence of staphylococcal canine pathogens resistant to multiple antimicrobial agents is a growing and urgent problem in veterinary practice. Antimicrobial peptides (AMPs) seem to be a promising alternative to conventional antibiotics. The aim of this in vitro study was to evaluate the antimicrobial activity of selected AMPs against pathogenic staphylococcal strains, including multidrug- and methicillin-resistant strains isolated from canine pyoderma cases. Seven antimicrobial peptides (aurein 1.2, CAMEL, citropin 1.1, protegrin-1, pexiganan, temporin A and uperin 3.6) synthesized by the 9-fluorenylmethoxycarbonyl (Fmoc) solid-phase method were tested. The minimal inhibitory and minimal bactericidal concentrations (MIC and MBC) were determined by the broth microdilution method. The study showed that analyzed AMPs exerted an extensive effect against canine pathogens, with the most active peptide being uperin 3.6. The tested AMPs were equally efficient against both resistant- and susceptible staphylococcal strains and were more efficient against *Staphylococcus pseudintermedius* than against *Staphylococcus aureus* strains. Our findings are particularly interesting from a clinical perspective, as they point to AMPs as potential therapeutic topical agents in canine pyoderma cases associated with antimicrobial resistance of staphylococci.

## 1. Introduction

Nowadays, the emergence of staphylococcal pathogens resistant to multiple antimicrobial agents is a growing public health threat. This ubiquitous and urgent clinical problem concerns not only human medicine, but also veterinary practice. In companion animals, canine skin infections constitute the main reason for antibiotics use [1], and consequently, may promote the development of resistance among canine bacteria [2,3]. Coagulase-positive staphylococci (CoPS), mostly *S. pseudintermedius* and *S. aureus*, are predominant pathogens in canine pyoderma [4,5]. The antimicrobial resistance of CoPS has a key role in the management of canine skin infections. The vast majority of those diseases have a chronic character with a tendency to recur, which is reflected by prolonged systemic antibiotic therapy, associated with an alarming increase in the proportion of antibiotic-resistant staphylococci, particularly methicillin-resistant staphylococci [3,5].

Resistance to methicillin is determined by modified protein, PBP2a, encoded by the *mecA* gene, and its new homologues (*mecB*, *mecC*, and *mecD*) located on a mobile genetic element known as the staphylococcal cassette chromosome (SCC*mec*). This cassette can be exchanged between various strains of the same species, or even between various staphylococcal species, and can carry genes encoding resistance to other antibiotics. Methicillin-resistant (MR) strains show resistance to all beta-lactam antibiotics, including antibiotics with beta-lactamase inhibitors and carbapenems. Furthermore, resistance to methicillin is frequently associated with resistance to other groups of antibiotics (macrolides, lincosamides, aminoglycosides, fluoroquinolones and sulfonamides) and determines multiple-drug resistance [6,7].

Antimicrobial peptides (AMPs) seem to be a perfect alternative to conventional antibiotics—not only due to their wide activity, but also to their low propensity to induce microbial resistance [8]. AMPs belong to a large family of cationic peptides that exert their bactericidal activity by destabilizing the bacterial membrane or increasing its permeability. Thus, they act quickly and selectively against bacteria at micromolar concentrations [9]. AMPs, as the next generation of antibacterials, are the object of rapidly-growing research interest and may also be promising antimicrobials for the treatment of staphylococcal skin infections in dogs, particularly where antibiotic therapy may be limited because of multidrug resistance [10].

A number of research groups worldwide continue research on the potential therapeutic application of AMPs, and some of these studies have already entered a clinical phase. AMPs were tested as treatments for meningococcal meningitis [11], local catheter-related infections [12], acne [13], oral mucosal infections [14] and diabetic foot syndrome [15]. Currently, two promising approaches are adopted for the treatment of canine infections, used in shampoos, foams and ear gels. These approaches involve plant extracts which promote the production of endogenous AMPs by the canine skin [16], and a cyclic β-sheet synthetic peptide (AMP2041), which exerts broad-spectrum antimicrobial activity [17,18]. In this study, we selected antimicrobial peptides with an established antibacterial activity in human infections and that have not been previously tested against canine staphylococcal pathogens. Selected by us, protegrin-1, like AMP2041, is a cyclic β-sheet peptide which demonstrates good antibacterial activity against a variety of human pathogens [19]. The aim of this in vitro study was to evaluate the antimicrobial activity of selected AMPs against pathogenic staphylococcal strains, including multidrug- and methicillin-resistant strains isolated from canine pyoderma cases. 

## 2. Materials and Methods

### 2.1. Bacterial Strains

Sixty-six well-characterized staphylococcal strains (60 *S. pseudintermedius* and 6 *S. aureus* strains) were selected from the archived previously described collection [4,20]. This study was conducted based on a retrospective analysis of staphylococcal canine strains isolated and archived at the Laboratory of Department of Medical Microbiology of MUG during routine clinical laboratory procedures. All samples were routinely collected by a veterinarian during the course of infection treatment or control visits, not specifically for this research. The animals were swabbed only after the owner’s consent was given, as we stated in the previous published study [4].

The strains were isolated from samples obtained by swabbing diseased sites by a veterinarian with a sterile cotton swab and were only taken from dogs with evident symptoms of infection (papules or pustules, dry or flaky patches of skin, hair loss and pruritus). 

All strains were differentiated for *S. pseudintermedius* using the PCR-RFLP method described by Bannoehr et al. [21]. The identity of *S. aureus* strains was verified based on the polymerase chain reaction (PCR) of the *S. aureus*-specific region of the thermonuclease gene, *nuc* [22].

The susceptibility of the selected strains to conventional antibiotics was determined by the disk diffusion method and interpreted for *S. pseudintermedius* according to the Clinical and Laboratory Standards Institute document VET01-A4 [23], and for *S. aureus* according to CLSI document M100-S25 [24]. The following drugs were used as representatives of the principal antimicrobial classes: amoxicillin, cefadroxil, cefoxitin (for prediction of methicillin-resistance in *S. aureus*), chloramphenicol, ciprofloxacin, clindamycin, doxycycline, erythromycin, gentamicin, sulfamethoxazole/trimethoprim, oxacillin (for prediction of methicillin-resistance in *S. pseudintermedius*) and tetracycline (Becton Dickinson, USA). Resistance to methicillin was additionally verified based on the detection of a *mecA* gene [25].

Staphylococcal strains were classified as multidrug-resistant (MDR) when they were not susceptible to at least one agent in three different classes of antimicrobials. Sixty examined *S. pseudintermedius* strains included thirty multidrug-resistant strains (MDRSP) and seven methicillin-resistant strains (MRSP). Six canine *S. aureus* strains, including three multidrug-resistant strains (MDRSA). *S. pseudintermedius* strains, including MDRSP (*n* = 30) and MRSP (7) strains, were resistant to: amoxicillin (61.6%), clindamycin (48.3%), erythromycin (45%), gentamicin (33.3%), chloramphenicol (26.6%), sulfamethoxazole/trimethoprim (25%), tetracycline (20%), cefadroxil (11.6%), oxacillin (11.6%) and ciprofloxacin (10%). Six *S. aureus* strains, including MDRSA (*n* = 3), were resistant to: amoxicillin (3/6), erythromycin (3/6), gentamicin (3/6), clindamycin (2/6), chloramphenicol (1/6), sulfamethoxazole/trimethoprim (1/6) and doxycycline (1/6). The following reference strains were used: *S. aureus* ATCC 6538 (MSSA), *S. aureus* ATCC 43300 (MRSA), *S. intermedius* PCM 2405. Both the reference- and clinical strains were stored at −80 °C in Tryptic Soy Broth (TSB, Becton–Dickinson, USA) supplemented with 15% glycerol.

### 2.2. Antimicrobial Peptides

In this study, seven AMPs were used: aurein 1.2, CAMEL (CA(1–7)M(2–9)), citropin 1.1, protegrin-1, pexiganan, temporin A and uperin 3.6 (Table 1). The peptides were synthesized by the 9-fluorenylmethoxycarbonyl (Fmoc) solid-phase method on Rink amide resin (Orpegen Peptide Chemicals GmbH, Heidelberg, Germany). All reactions were induced using a heating clamp HC60 (Kamush®, Gdansk, Poland), which increases the efficiency of synthesis owing to the heating of the reaction vessel. Reagents were dissolved in *N,N*-dimethylformamide (DMF) in a fourfold excess based on the resin (Fmoc-AA: DIC: OxymaPure, 1:1:1, mol/mol). Double Fmoc deprotection was accomplished by adding 20% piperidine to DMF for 2 minutes (2 × 2 min), whereas the coupling steps were performed for 15 minutes, both at 60 °C (the coupling of cysteine residue at 50 °C). The peptides were cleaved from the resin using one of the following mixtures: (A)—trifluoroacetic acid (TFA, Apollo Scientific, Denton, UK), 1,2-ethanedithiol (EDT, Merck, Darmstadt, Germany), phenol, triisopropylsilane (TIS, Acros Organics, Geel, Belgium) and water (92:2:2:2:2 v/v/w/v/v); (B)—TFA, TIS, phenol and water (94:2:2:2 v/v/w/v); (C)—TFA, TIS and water (96:2:2 v/v/v). Mixture (A) was used with peptides containing cysteine residues, mixture (B) was used with CAMEL (CA(1–7)M(2–9)), and mixture (C) was used for the remaining peptides. Cleavage was accomplished within 1 h while the mixtures were being stirred. Moreover, protegrin-1 was synthesized with orthogonally protected cysteine residues (Fmoc-L-Cys(Trt)-OH for C8 and C13, and Fmoc-L-Cys(Acm)-OH for C6 and C15). Protegrin-1 was dissolved in water and oxidized to form disulfide bridges between the appropriate cysteine residues (8–13 and 6–15) through oxidation with iodine. The progress of the reaction was monitored by LC-MS. A Waters Alliance e2695 RP-HPLC system with Waters 2998 PDA and Acquity QDA detectors was used. Peptides were purified using reversed-phase high-performance liquid chromatography (RP-HPLC) on a Phenomenex Gemini-NX C18 column (21.20 × 100 mm, 5.0 µm particle size and 110 Å pore size) with UV detection at 214 nm. Acetonitrile and water, both containing 0.1% of TFA, were used for the mobile phase. The crude peptides were eluted with a linear 20%–70% acetonitrile gradient in deionized water for over 50 min at a flow rate of 10.0 mL/min. The purity and identity of the peptides were confirmed with an LC-MS analysis. The pure fractions (>95%, by an HPLC analysis) were collected and lyophilized.

### 2.3. In Vitro Susceptibility Testing

The antimicrobial susceptibility of each strain to the tested peptides was determined using the minimal inhibitory concentrations (MIC) microdilution method as recommended by the Clinical Laboratory Standards Institute (CLSI) guidelines. Briefly, the staphylococcal strains were grown overnight on a Columbia blood agar (Graso Biotech, Poland) at 37 °C. Isolated colonies were suspended in saline solution and adjusted to a 0.5 McFarland standard. The bacterial suspension was diluted ~ 1:100 to a final concentration of 1 × 10^6^ colony forming units (cfu/mL). Two-fold serial dilutions of each tested peptide in Mueller–Hinton Broth 2 (Sigma–Aldrich) were added (100 µL) to polypropylene 96-well plates containing bacterial inoculum (100 µL) and were incubated at 37 °C for 18 h. All clinical and reference strains were tested in duplicate. Bacterial growth was assessed visually after incubation and MIC was recorded as the lowest drug concentration at which an observable growth was inhibited. The MIC_50_ and MIC_90_ values were defined as the lowest concentrations of peptides at which 50% and 90% of the strains were inhibited, respectively.

The minimal bactericidal concentration (MBC) was determined in a sample taken from each test tube in which no growth was observed in the MIC assay. The loopful (10 µl) of the tested sample was transferred to Triptic Soy Broth (TSB, BD Difco) and incubated at 37 °C for 48 h. MBC was taken as the lowest concentration of each peptide at which staphylococcal colonies were no longer viable on the subculture. 

### 2.4. Statistical Analysis

Statistical characteristics of MIC/MBC values were presented as medians and lower- and upper quartiles and ranges. The significance of between-group differences in MIC/MBC was verified with the Mann–Whitney U-test. The differences were considered significant at *p* < 0.05. All calculations were carried out with a Statistica 10 package (StatSoft, Tulsa, OK, USA).

## 3. Results

**Figure 1:**
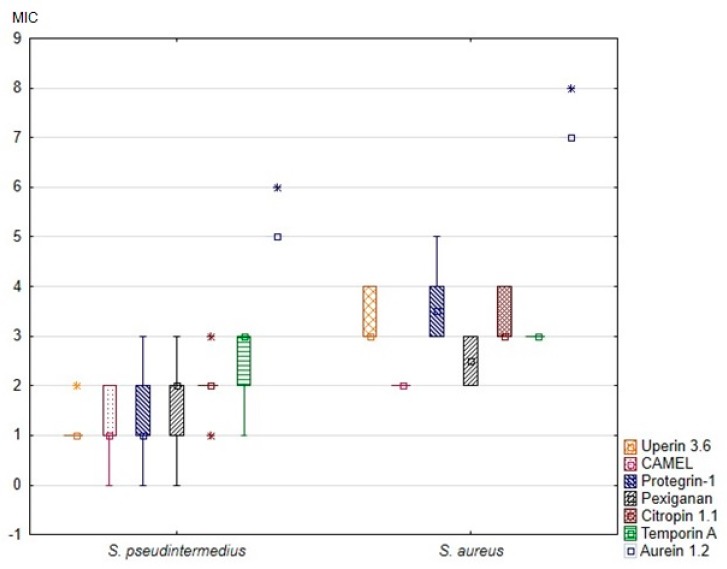
Minimal inhibitory concentrations (MIC) of the tested antimicrobial peptides against canine *S. pseudintermedius* and *S. aureus* strains. The results are presented as medians (points), interquartile ranges (boxes), ranges (whiskers) and outliers (asterisks). Minimal inhibitory concentrations on the Y axis are shown as log2(x).

All tested peptides were active against all reference- and clinical staphylococcal strains, with both median MICs and median MBCs ranging from 2 µg/mL to 128 µg/mL. Most MBCs corresponded to the same or double the dilution concentration as the respective MICs. The MIC and MBC of each peptide against the tested strains are summarized in Table 2 and Table 3. The MICs and MBCs against *S. pseudintermedius* and *S. aureus* differed considerably. Specifically, the MICs of all tested peptides against *S. aureus* were significantly higher than the MICs against *S. pseudintermedius* (Figure 1). Additionally, the MBCs of all peptides but CAMEL were significantly higher in the case of *S. aureus* (Table 2).

No significant differences in the susceptibility to AMPs were found between the susceptible- and multidrug-resistant strains, nor between the methicillin-resistant and -susceptible strains (*p* < 0.05).

## 4. Discussion

In this study, we selected antimicrobial peptides with an established antibacterial activity in human infections that have not been previously tested against canine staphylococcal pathogens, including *S. pseudintermedius* and *S. aureus* strains, isolated from cases of canine pyoderma.

Our study compared the activity of the tested compounds against both susceptible- and multi-resistant canine strains. Interestingly, no differences in terms of sensitivity to the AMPs were found between the susceptible- and multi-resistant strains, nor as between the methicillin-resistant and -susceptible strains. This certainly represents an advantage of AMPs, since MR-associated infections are usually more difficult to treat with antibiotic therapy than those caused by MS strains. Our results demonstrate that AMPs work equally for *S. pseudintermedius,* irrespective of their drug resistance status. These findings are consistent with the results of previous studies [26].

Furthermore, we demonstrated that the strains of CoPS, belonging to different antibiotic patterns and isolated from different dogs, showed the same levels of susceptibility to the tested antimicrobial peptides. This indicates that mechanisms of antimicrobial resistance did not affect the examined peptides. These findings support the hypothesis that AMPs might be promising topical drug molecules to treat canine pyoderma. The topical antimicrobial treatment of canine superficial pyoderma is favourable to systemic treatment, as very high concentrations can be reached at the site of infection and as it minimizes resistance development [27]. Additionally, the relatively low MICs and MBCs of the tested peptides are potentially advantageous when a high concentration is applied topically.

Uperin 3.6 turned out to be the most efficient agent against the tested *S. pseudintermedius* strains in our study. Uperin 3.6 is a wide-spectrum, 17-residue antimicrobial peptide, isolated from the Australian toadlet, *Uperoleia mjobergii*. It is one of the most potent membrane-active antimicrobial peptides isolated from amphibians [28]. The range of MICs for uperin 3.6 against our staphylococcal strains was similar to that in a study conducted by Giacometti on human *S. aureus* strains [29]. Unlike in studies of human *S. aureus* strains isolated from patients with cystic fibrosis and skin infections, uperin 3.6 turned out to be the most effective AMP against canine staphylococci [26,30]. Compared with previous studies—in which the MICs for uperin varied between 128 µg/mL and 256 µg/mL—in our experiment, the MICs for all *S. pseudintermedius* strains were significantly lower. Canine strains of *S. aureus* were considerably less sensitive to uperin than to *S. pseudintermedius*. As a typical human pathogen, *S. aureus* is a markedly less common etiological factor in canine skin infections. Given the results of our present study, and the fact that *S. pseudintermedius* is the most common cause of skin infections in dogs, uperin 3.6 could be considered as a potential antibacterial agent, especially in cases associated with the antimicrobial resistance of staphylococci.

The results on the activity of the remaining peptides revealed that synthetic AMPs, such as CAMEL, protegrin-1 and pexiganan, were highly effective against *S. pseudintermedius* strains and less effective against *S. aureus* strains. Pexiganan, a synthetic analogue of magainin II, deserves attention not only for its anti-staphylococcal activity, but also in view of its efficiency in clinical trials. An in vivo study of patients with mildly infected diabetic foot ulcers demonstrated that topical pexiganan was efficacious in terms of clinical- and microbiological improvement and wound healing rates [15].

## 5. Conclusions

In conclusion, the tested AMPs were shown to be equally efficient antimicrobial agents against resistant- and susceptible pathogenic staphylococcal strains associated with canine pyoderma. Our findings are particularly interesting from a clinical perspective, as a starting point from which to perform in vivo experiments to estimate the usefulness of these peptides as topical drug molecules for the treatment of canine pyoderma.

## Figures and Tables

**Table 1:** Amino acid sequences of tested antimicrobial peptides.

Peptide	Amino Acid Sequence
aurein 1.2	GLFDIIKKIAESF-NH_2_
CAMEL	KWKLFKKIGAVLKVL-NH_2_
citropin 1.1	GLFDVIKKVASVIGGL-NH_2_
pexiganan	GIGKFLKKAKKFGKAFVKILKK-NH2
* protegrin-1	RGGLCYCRGRFCVCVGR-NH_2_
temporin A	FLPLIGRVLSGIL-NH_2_
uperin 3.6.	GVIDAAKKVVNVLKNLF-NH_2_

* protegrin 1: disulfide 8–13 and 6–15.

**Table 2:** Minimal inhibitory concentrations (MIC) and minimal bactericidal concentrations (MBC) of the tested antimicrobial peptides against the canine *S. pseudintermedius* (I) and *S. aureus* (II) strains.

Parameter		Uperin 3.6		CAMEL		Protegrin-1		Pexiganan		Citropin 1.1		Temporin A		Aurein 1.2	
		I	II	I	II	I	II	I	II	I	II	I	II	I	II
**M** **I** **C**	Median (IQR)	2 (2–2)	8 (8–14)	2 (2–4)	4 (4–4)	2 (2–4)	12 (8–16)	4 (2–4)	6 (4–8)	4 (4–4)	8 (8–14)	8 (4–8)	8 (8–8)	32 (32–32)	128 (128–128)
	MIC_50_	2	8	2	4	2	8	4	4	4	8	8	8	32	128
	MIC_90_	2	16	4	4	4	32	4	8	4	16	8	8	32	256
	*p*-value *	<0.001		0.014		<0.001		0.006		<0.001		0.041		<0.001	
**M** **B** **C**	Median (IQR)	2 (2–4)	16 (16–16)	4 (2–4)	4 (4–4)	4 (2–4)	12 (8–16)	4 (2–4)	8 (8–8)	4 (4–4)	12 (8–16)	8 (8–8)	8 (8–14)	64 (64–64)	128 (128–224)
	MBC_50_	2	16	4	4	4	8	4	8	4	8	8	8	64	128
	MBC_90_	4	16	4	4	8	32	8	8	8	16	8	16	64	256
	*p*-value *	<0.001		0.055		<0.001		<0.001		<0.001		0.045		<0.001	

* Mann–Whitney U test, IQR—interquartile range.

**Table 3:** Detailed comparison of MIC values of tested antimicrobial peptides against canine staphylococci.

Peptide	Species	MIC [µg/mL]									Range
		1	2	4	8	16	32	64	128	≥256	[µg/mL]
Uperin 3.6	*S. pseudintermedius* (MSSP)		50	3							0.5–64
	*S. pseudintermedius* (MRSP)		6	1							0.5–64
	*S. aureus*				4	2					0.5–64
	*S. aureus* ATCC 6538					1					0.5–64
	*S. aureus* ATCC 43300					1					0.5–64
	*S. intermedius* PCM 2405				1						0.5–64
Protegrin-1	*S. pseudintermedius*(MSSP)	1	28	21	3						0.5–64
	*S. pseudintermedius* (MRSP)		4	2	1						0.5–64
	*S. aureus*				3	2	1				0.5–64
	*S. aureus* ATCC 6538					1					0.5–64
	*S. aureus* ATCC 43300			1							0.5–64
	*S. intermedius* PCM 2405				1						0.5–64
CAMEL	*S. pseudintermedius* (MSSP)	2	25	26							0.5–32
	*S. pseudintermedius* (MRSP)		6	1							0.5–32
	*S. aureus*			6							0.5–32
	*S. aureus* ATCC 6538			1							0.5–32
	*S. aureus* ATCC 43300			1							0.5–32
	*S. intermedius* PCM 2405		1								0.5–32
Pexiganan	*S. pseudintermedius*(MSSP)	1	23	26	3						0.5–32
	*S. pseudintermedius* (MRSP)			6	1						0.5–32
	*S. aureus*			3	3						0.5–32
	*S. aureus* ATCC 6538				1						0.5–32
	*S. aureus* ATCC 43300				1						0.5–32
	*S. intermedius* PCM 2405			1							0.5–32
Citropin 1.1	*S. pseudintermedius* (MSSP)		1	49	3						0.5–32
	*S. pseudintermedius* (MRSP)			3	4						0.5–32
	*S. aureus*				4	2					0.5–32
	*S. aureus* ATCC 6538				1						0.5–32
	*S. aureus* ATCC 43300					1					0.5–32
	*S. intermedius* PCM 2405			1							0.5–32
Temporin A	*S. pseudintermedius* (MSSP)			24	29						0.5–32
	*S. pseudintermedius* (MRSP)			1	6						0.5–32
	*S. aureus*				6						0.5–32
	*S. aureus* ATCC 6538				1						0.5–32
	*S. aureus* ATCC 43300				1						0.5–32
	*S. intermedius* PCM 2405			1							0.5–32
Aurein 1.2	*S. pseudintermedius* (MSSP)						49	4			8–256
	*S. pseudintermedius* (MRSP)						7				8–256
	*S. aureus*								5	1	8–256
	*S. aureus* ATCC 6538								1		8–256
	*S. aureus* ATCC 43300								1		8–256
	*S. intermedius* PCM 2405							1			8–256
